# Positional transversal release is effective as stretching on range of movement, performance and balance: a cross-over study

**DOI:** 10.1186/s13102-022-00599-8

**Published:** 2022-11-30

**Authors:** Ewan Thomas, Salvatore Ficarra, Antonino Scardina, Marianna Bellafiore, Antonio Palma, Nemanja Maksimovic, Patrik Drid, Antonino Bianco

**Affiliations:** 1 Sport and Exercise Sciences Research Unit, Department of Psychology, Educational Science and Human Movement, University of Palermo, Via Giovanni Pascoli 6, 90144 Palermo, Italy; 2 Faculty of Sport and Physical Education, University of Novi Sad, 21000 Novi Sad, Serbia

**Keywords:** Stretching, ROM, Long jump, Balance, Positional transversal release

## Abstract

**Background:**

The aim of this study was to compare the positional transversal release (PTR) technique to stretching and evaluate the acute effects on range of movement (ROM), performance and balance.

**Methods:**

Thirty-two healthy individuals (25.3 ± 5.6 years; 68.8 ± 12.5 kg; 172.0 ± 8.8 cm) were tested on four occasions 1 week apart. ROM through a passive straight leg raise, jumping performance through a standing long jump (SLJ) and balance through the Y-balance test were measured. Each measure was assessed before (T0), immediately after (T1) and after 15 min (T2) of the provided intervention. On the first occasion, no intervention was administered (CG). The intervention order was randomized across participants and comprised static stretching (SS), proprioceptive neuromuscular facilitation (PNF) and the PTR technique. A repeated measure analysis of variance was used for comparisons.

**Results:**

No differences across the T0 of the four testing sessions were observed. No differences between T0, T1 and T2 were present for the CG session. A significant time × group interaction for ROM in both legs from T0 to T1 (mean increase of 5.4° and 4.9° for right and left leg, respectively) was observed for SS, PNF and the PTR. No differences for all groups were present between T1 and T2. No differences in the SLJ and in measures of balance were observed across interventions.

**Conclusions:**

The PTR is equally effective as SS and PNF in acutely increasing ROM of the lower limbs. However, the PTR results less time-consuming than SS and PNF. Performance and balance were unaffected by all the proposed interventions.

## Introduction

In recent years, the interest in stretching exercises has grown and the possible effects on the cardiovascular and nervous system [1, 2] and its role in injury prevention and rehabilitation [3, 4], sporting enhancement [5], and balance have been investigated. Although, the main aim of stretching still remains to improve range of movement (ROM) of a joint and increase the flexibility of muscles and tendons [4, 6].

The effects of stretching in increasing ROM have been attributed to four main mechanisms, involving sensory, neural and structural (muscle and tendon) adaptations [7]. Differences in adaptational mechanisms may be observed after acute or chronic interventions [7, 8]. Although changes in stretch tolerance are those most frequently observed regardless of intervention length [9, 10]. Among the stretching methodologies, the most common and most studied are static (SS), proprioceptive neuromuscular facilitation (PNF) and dynamic stretching [11]. Acute responses on ROM, indicate that stretching improves ROM in a time-dependent manner [5, 12]. Effects which seem to be independent of stretch typology [5, 13].

Stretching has been also deeply investigated in regard to its effects on performance as power, strength and speed [5, 14]. Differently from ROM, the acute effects seem to strongly depend on stretch typology. Bouts of SS are generally observed to negatively affect strength or power performance if applied immediately before the activities [15, 16]. Conversely, dynamic stretching may enhance power activities as jumping [15]. There is still debate if stretch duration is crucial in performance hampering since some studies have observed decreased performance parameters after a single set of short duration stretching [17, 18] while others have observed such effects only after sets of longer duration (above 60 s) [14]. The performance hampering effects observed as a consequence of stretching are attributed to reduced neural drive by a reduction of the excitability of α-motor neurons [19]. This reduction in motor neuron excitability is ascribed to a reduction of persistent inward current (PIC), a depolarizing current with the property of amplifying the synaptic input–output of α-motor neurons [20] allowing higher depolarization frequencies, necessary for maximal force production [21]. Therefore, reduced PIC will determine reduced synaptic output with a reduction in the force output of muscles. These effects have been only observed after passive stretching [19], which may explain the discrepancy in outcomes observed after dynamic stretching.

Another investigated aspect of stretching is its effect on balance. However, univocal conclusions could not be drawn since in some cases balance increased [22, 23], in others decreased [24, 25] or no effects [26, 27] were observed regardless if measures of static or dynamic balance were assessed. Differences in outcomes were identified pertaining either to stretch typology [24] or the screened population [22]. Untrained individuals or those with impaired balance may benefit from performing stretching. While longer bouts of static stretching may reduce the evaluated balance parameters. Most importantly, high heterogeneity among the evaluation tests adopted can be observed across studies (i.e. single leg stance, stabilometric platforms, swinging platforms, etc.), which could be an additional factor influencing the discrepant results.

Other approaches different from stretching have been adopted for ROM improvement. One of these is myofascial release [28] (MFR, a term which incorporates a wide variety of manual techniques applied to muscles and fascia [29]), usually employed by physical therapists. These techniques when compared to stretching, have shown to provide equally effective results on ROM improvement [30–33]. Advantages of MFR techniques are that (1) they do not involve joint movement, allowing to be specific on soft tissues, therefore limiting joint dysfunction when present (reason by which many physical therapists adopt them) [31] and (2) it generally does not require much time to achieve a result (90–120 s) [34]. Neurophysiological adaptations seem to acutely drive the increased ROM observed [29]. These are thought to involve the Golgi reflex arc mediated by Golgi tendon organs and Ruffini and Pacini receptors which are sensitive to pressure [35]. The application of manual pressure to deep fascia decreases motor neuron excitability and may lead to reduced muscular tension allowing greater ROM [36]. However, conclusive mechanisms explaining the increased ROM observed are still to be determined.

Effects of MFR have been also studied on performance measures of power, force development or agility and static and dynamic balance, however, these usually result unaffected by the application of the techniques [29, 37–39]. Since MFR techniques have the potential to improve ROM without impairing performance or balance parameters as observed after SS, it has been speculated that MFR could lead to improved muscle efficiency [38] supporting its use in sporting and rehabilitation environments.

Within this context, a hybrid technique incorporating components of SS and MFR is here proposed. This novel technique, the Positional Transversal Release (PTR), involves a passive stretch of the targeted muscle, followed by manual stimulation of the musculotendinous junction of the stretched muscle. Therefore, with this study, we aim to examine and compare this novel approach to traditional stretching and determine the acute effects on measures of ROM, performance and balance in healthy individuals.

## Methods

### Design

This study aimed to examine and compare a new technique to two traditional stretching approaches and determine the acute effects on ROM of the lower limb, jumping performance and dynamic balance in healthy individuals. A crossover research model was adopted in which each participant was tested on four occasions. A 1 week wash-out period was provided between subsequent testing sessions. The participants were tested in a university laboratory environment. On each visit, each participant was tested in three occasions (described in the following paragraphs). Each visit lasted ⁓ 1 h.

The study was conducted in accordance with the deontological norms laid down in the Helsinki Declaration and the European Union recommendations for Good Clinical Practice and the Ethical Standards in Sport and Exercise Science Research [40]. The study was approved by the University bioethical committee (protocol n°65/2021).

### Participants

Thirty-two participants (mean ± SD: 25.34 ± 5.56 years; 68.77 ± 12.54 kg; 172 ± 8.83 cm) of which 13 females and 19 males participated in this study. The participants were recruited from a population of university students. All participants provided written informed consent. Each participant was informed about the procedures, risks and benefits, of participating in this research but not regarding the research hypothesis. Subjects were eligible of inclusion if healthy, while no limitations regarding sex or age were applied. Subjects were excluded if injured or complained of musculoskeletal or neurological constraints. Competitive athletes were also excluded.

### Procedures

**Fig. 1:**
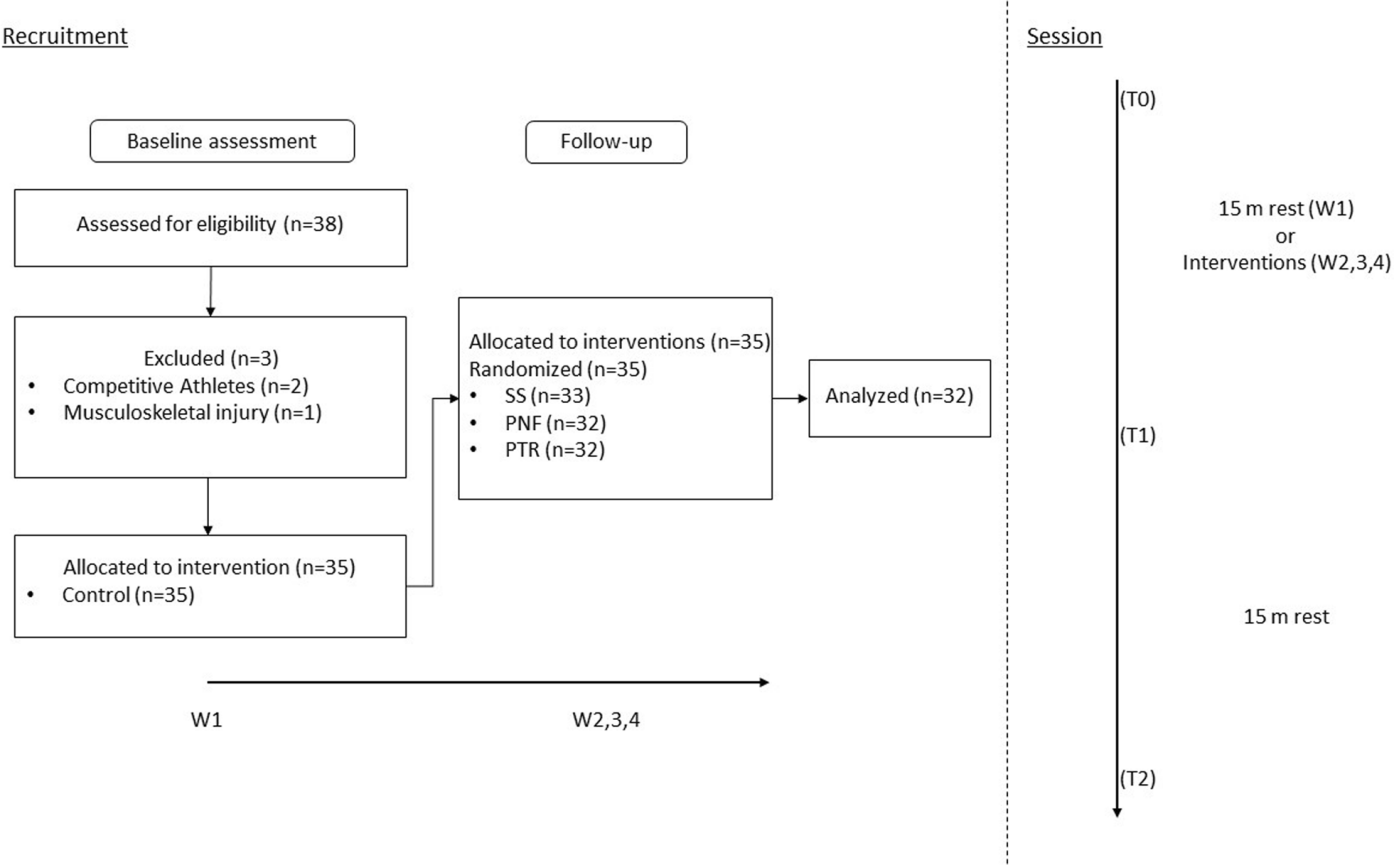
Schematic representation of recruitment and assessment procedures. The left panel shows the recruitment procedures, assessment for eligibility and allocation to intervention groups. The right panel shows the time course of the assessment procedures for each session. W1: Week 1 (control measurements); W2,3,4: Weeks 2, 3 and 4 (subsequent evaluations, intervention measurements); T0: baseline evaluation; T1: post intervention or post-15 m rest evaluation; T2: evaluation after 15 m-rest post T1; SS: static stretching; PNF: proprioceptive neuromuscular facilitation; PTR: positional transversal release

A total of thirty-five participants were initially recruited. Recruitment procedures were carried out through social media advertisement. Each participant therefore voluntarily participated. Each participant after being informed about study procedures had to complete a questionnaire regarding physical activity level (IPAQ) [41]. Based on individual responses the participants were classified as moderately active. All the subjects were asked to maintain their normal physical activities during the study but to refrain from any form of stretching exercise. Each participant had to attend the laboratory on four occasions, 1 week apart from each visit. The first visit served as a control, while on the following visits each participant had to undergo a different intervention. The administration order of the interventions was randomized for each participant. On each visit, each participant underwent a passive straight leg raise test (PSLR) for both lower limbs to assess ROM, the standing long jump (SLJ) to assess a measure of performance and the Y-balance test (YBT) for dynamic balance. Each test was assessed at baseline (T0), immediately after the stretching intervention (T1) and fifteen minutes after the end of the intervention (T2). During the first visit (control session), the participants sat on a chair for fifteen minutes between T0 and T1. Of the thirty-five participants initially recruited, three did not attend the laboratory for all planned consecutive assessments, therefore these were not included in the final analysis. Recruitment and procedures are presented in Fig. 1.

### Measures

To ensure a high scientific standard, all measures were assessed by the same investigator, which was blinded regarding the proposed interventions. T0 and subsequent T1 and T2 were implemented at the same time of the day for each participant. To avoid any bias, measurements were performed without warm-up in the following order:

(1) PSLR, (2) YBT and (3) SLJ. The YBT and the SLJ were sampled three times and only the best measure was retained for investigation. The PSLR was assessed once, in order to avoid a repetition-dependent effect on ROM [42].

*Range of movement* Hip flexion was measured with a Gyko inertial sensor system (Microgate, Bolzano, Italy) [43]. Using Bluetooth 4.0, information was streamed to a computer with dedicated software (Gyko RePower; Microgate, Bolzano, Italy). A standard procedure for the PSLR was adopted. Participants were first instructed to lie supine on a medical bed. The Gyko was strapped at the level of the distal end of the femur of the tested leg. Inelastic straps were used to fix the contralateral limb to the medical bed. The tested leg was then passively lifted in full extension to the limit of the available ROM, or the point the participants started to feel pain or discomfort [44]. The procedure was repeated for both limbs. The testing order of the limbs was randomized across participants.

*Y-balance test* A standard procedure for the YBT was adopted using a YBT kit (Functional Movement Systems®, Chatham, USA) [45] for dynamic balance evaluation. The kit is composed of a centralized platform and three pipes which connect to the platform. Each pipe is oriented in a different direction. The pipes are marked with 1 cm increments over which a moveable reach indicator is positioned. Participants were asked to move each reach with their leg as far as possible along each pipe while standing with the other leg on the central platform. The procedure was then repeated with the contralateral leg. In order to identify a “balance index” for each leg, leg length was calculated (From the anterior superior iliac spine to the lower margin of the lateral malleolus). The balance index was calculated by summing the distances covered over the three pipes and dividing this measure by three, to obtain an absolute mean distance. The absolute mean distance was divided by leg length and the result was finally multiplied by 100.

*Standing long jump* A measure of performance of the lower limbs was collected through the SLJ. Each participant was asked to stand behind a line, with the feet at shoulder width and the toes touching, but not crossing the line. At the “go” of the investigator, the participants had to jump as far as possible. The participants were allowed to swing their arms during the jump. The jumping distance was measured through a tape measure, from the take-off line to the heels of the participant [46].

### Interventions

All interventions were carried out by the same investigator (kinesiologist and manual therapist with ⁓ 5 years of experience). Each subject had to undergo all the proposed interventions and was informed about the stretching procedure prior to its administration. The stretching procedures were intended to target the hamstring muscles.

The static stretching (SS) intervention consisted of 8 sets of a 30-s passive stretch with 30-s rest between each set. Each participant was asked to sit on a mat, with the knees straight and the feet touching a wall with the feet in dorsiflexion at 90°. Without flexing the knees, each participant was instructed to flex their trunk over the hips in order to reduce the hip flexion angle. The investigator helped the participant towards the maximum tolerable trunk flexion angle and maintained the participant’s position for the duration of the planned stretch by positioning his hands on the lower back of the participant. This protocol was chosen since it is frequently adopted in studies evaluating the acute effects of stretching [47].

An equated volume protocol concerning the stretch phase was adopted for the Proprioceptive Neuromuscular Facilitation (PNF) intervention. The protocol consisted of 8 sets of stretching with a 30-s rest between each set. The procedure was the same as that of the SS. Once the participant reached the maximum tolerable flexion angle, the passive stretch was maintained for 10-s, followed by an isometric contraction against the investigator’s hands for 6-s, followed by a 4-s post-isometric relaxation phase. This procedure was repeated 3 times for each set.

**Fig. 2:**
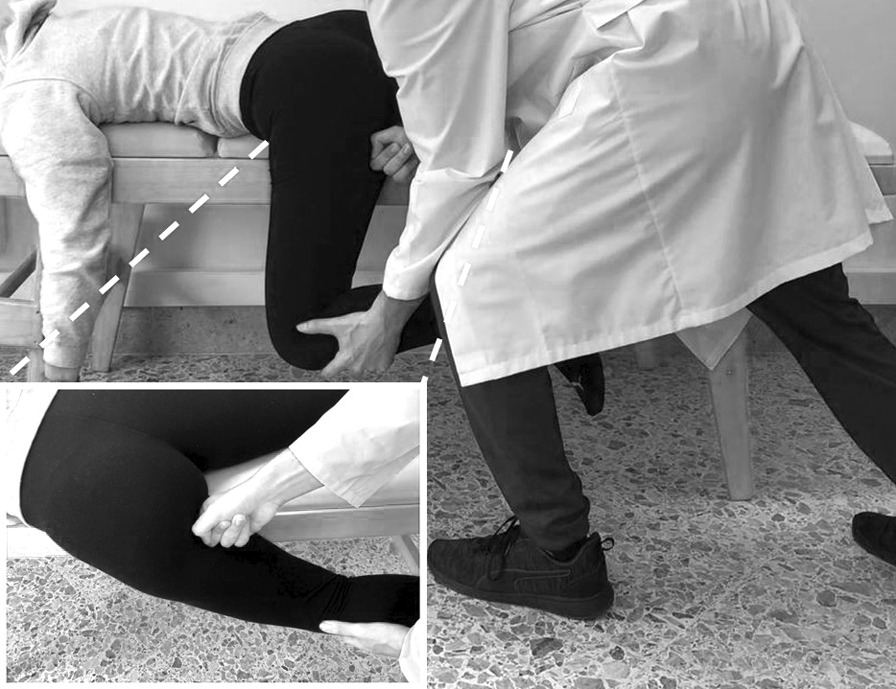
Representation of the Positional Transversal Release technique. The main scheme depicts the positioning of the participant and positioning of the operator. The box on the left lower aspect of the scheme depicts the positioning of the hand of the operator on the myotendinous junction of the hamstring muscle of the participant

The positional transversal release (PTR) consisted of 1 to 2 mechanical stimulations of the proximal insertion of the hamstring muscles at the level of the myotendinous junction (MTJ). This was defined as an area ⁓ 5 cm below the ischial tuberosity. Each participant was instructed to lie prone on the edge of a medical bed, with one limb hanging from the medical bed. The investigator with one arm passively accompanied the leg of the participant towards a sub-maximal hip flexion (stretch phase). No pain or discomfort had to be perceived by the participant. The investigator positioned his other hand, more specifically his knuckle, on the medial side of the hamstring’s MTJ [48, 49] and through a rapid motion, in a transversal direction (compared to the hamstring muscle fibres), stimulated the MTJ (Fig. 2). During hand positioning, the investigator manually appraised the tension of the hamstring’s MTJ. Subsequently to the application of the PTR technique, the investigator re-appraised the hamstrings muscle tone and if a reduction was manually perceived the procedure was repeated on the contralateral limb. Otherwise, one additional stimulation was provided. No additional stimulations were provided, regardless if the perceived tone resulted or not decreased.

### Statistical analysis

A priori sample size calculation was performed through G-power (G*Power version 3.1.9.4, ES = 0.3, 1 − β = 0.80, α = 0.05) which defined as 32 the minimum number of participants required for the planned research model. Data are presented as means and standard deviations. Inferential statistics were carried out with Jamovi (The jamovi project (2021). jamovi (Version 1.8.0.1) [Computer Software]. Retrieved from https://www.jamovi.org). A Shapiro–Wilks test was performed to identify the normality of the distribution of all parameters. Repeated Measures Analysis of Variance (ANOVA) was performed for each measure using a 3 × 4 model to identify time and group interactions. Post-hoc Bonferroni corrections were performed to identify differences between the assessments for each group. Graphs were created with GraphPad Prism8 (GraphPad Software, San Diego, CA). An alpha level of p < 0.05 was defined for the statistical significance of all the tests.

## Results

Of the 35 participants initially enrolled, 32 completed all the evaluations (three participants did not show up for subsequent evaluations, Fig. 1) therefore, data from 32 participants were analysed.

No significant differences were observed when comparing the T0 across the 4 weeks for all tests (No differences between the control session and interventions at T0). No significant differences were observed for the control measures across time (T0, T1 and T2).

**Fig. 3:**
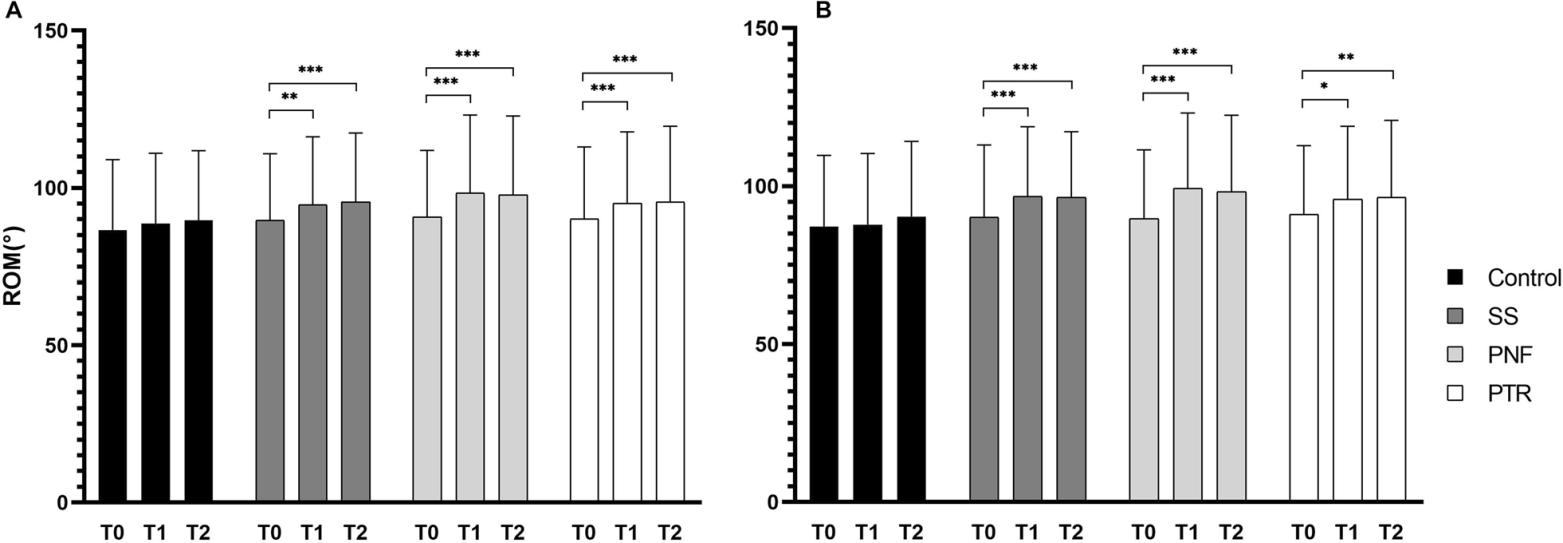
ROM of the lower limbs. **A** shows measures pertaining to the left leg. **B** shows measures pertaining to the right leg. ROM: range of movement; SS: static stretching; PNF: proprioceptive neuromuscular facilitation; PTR: positional transversal release. * p < 0.05; ** p < 0.01; *** p < 0.001

When considering the interventions, a significant time × group interaction was observed for ROM (Right leg F = 4.62; p = 0.0002, Left leg F = 2.64; p = 0.017). Post-hoc analysis revealed increases from T0 to T1 for SS, PNF and PTR. In all groups, ROM remained elevated in T2. No differences were observed for any intervention between T1 and T2. No differences were observed across interventions during the same time point evaluations (Table 1 and Fig. 3).

**Table 1:** The effects of stretching on variables of ROM, Jumping performance and Balance

	Control			SS			PNF			PTR			ANOVA
	T0	T1	T2	T0	T1	T2	T0	T1	T2	T0	T1	T2	
*ROM* (°)													
PSLR R	87.1 ± 22.6	87.8 ± 22.5	90.3 ± 23.8	90.1 ± 22.9	96.7 ± 22.1a	96.4 ± 20.8a	89.7 ± 21.7	99.3 ± 23.8a	98.2 ± 24.2a	91.1 ± 21.7	95.8 ± 23.1a	96.5 ± 24.3a	< 0.001*
PSLR L	86.7 ± 22.4	88.7 ± 22.3	89.4 ± 25.3	89.7 ± 21.1	94.6 ± 21.6a	95.6 ± 21.8a	90.8 ± 21.1	98.4 ± 24.8a	97.8 ± 25.1a	90.1 ± 22.9	95.1 ± 22.7a	95.6 ± 24.0a	0.017*
*Performance* (cm)													
SLJ	167.0 ± 34.5	168.7 ± 35.4	167.3 ± 36.4	171.9 ± 36.5	173.1 ± 37.7	175.0 ± 35.0	172.7 ± 35.1	167.5 ± 38.7	170.0 ± 36.0	172.0 ± 38.1	173.3 ± 37.4	174.0 ± 39.7	0.158
*Balance* (%)													
YBT R	93.2 ± 7.5	94.9 ± 7.7	96.0 ± 7.6	97.1 ± 6.6	97.4 ± 6.0	97.4 ± 6.5	96.9 ± 6.5	97.9 ± 7.0	98.0 ± 7.5	97.0 ± 6.7	97.6 ± 6.8	98.0 ± 6.8	0.678
YBT L	92.5 ± 9.2	94.4 ± 7.0	95.4 ± 7.8	96.5 ± 6.7	97.3 ± 6.7	97.4 ± 6.5	96.1 ± 6.9	97.0 ± 7.1	97.4 ± 6.8	97.0 ± 6.6	97.2 ± 6.9	97.4 ± 7.1	0.605

Data are presented as means ± SD; T0: baseline evaluation; T1: post-test assessment; T2: assessment 15 m post-test; PNF: proprioceptive neuromuscular facilitation; PTR: positional transversal release; ANOVA: group × time interaction

a Post-hoc difference with T0

*Significant *p* < 0.05

No time × group interactions were observed for the SLJ (F = 1.53; p = 0.158) and the YBT (Right F = 0.57; p = 0.678, Left F = 0.68; p = 0.605).

## Discussion

The aim of the present investigation was to examine and compare the acute effects of a novel technique to two stretching techniques applied to the hamstring muscles on ROM of the lower limbs, jumping performance and dynamic balance. Our results indicate that all three interventions were equally effective in increasing ROM, while no acute effects were observed for jumping performance and dynamic balance.

The acute ROM increases observed as a consequence of stretching are generally attributed to a reduction in sensation [7], which may either reflect a psychological alteration or the willingness of each participant to tolerate greater torque since these expect that following stretching interventions an increase in ROM should occur. The mechanisms attributed to increased ROM after MFR are usually ascribed as either Golgi tendon organs mediated or by Ruffini and Pacini mechanoreceptors reflexes [50]. Despite these mechanisms had been also traditionally attributed to stretching, more recent evidence does not support these assumptions, suggesting that modulation of pain sensation occurs [51, 52]. In this context, we tried to develop a technique which incorporated both aspects in order to improve the sought ROM. The first aspect of the PTR is the lengthening position, which may influence the participant’s sensation in a similar-stretching manner. The second aspect is the mechanical and transversal stimulation of the MTJ.

It is known that the MTJ is rich in mechanoreceptors, Golgi tendon organs on the muscular side and Pacini receptors on the tendinous side [36, 53]. These latter, which can be also found in the dermal layer, are sensitive to rapid pressure changes and vibration. In addition, Ruffini receptors can be found in ligaments and dermal tissues which are usually subjected to stretching and are particularly responsive to tangential forces [54]. Different experimental investigations have found interactions between these cutaneous receptors and proprioception [55–57] and subsequent applied research supports the inclusion of cutaneous stimuli, such as vibration, for ROM improvement [58–60]. The mechanical stimulation provided tangentially to the MTJ aims to stimulate both Pacini (through the rapid movement which mimics a high-frequency vibratory stimulus) and Ruffini receptors (through the cutaneous transversal direction). In a review by Proske and Gandevia [53], the authors describe that these receptors may provide positional sense by acting as “limit detectors”, which would suggest that alteration of afferent signals by these receptors may alter the proprioceptive positional limit of a joint. However, there are no studies evaluating if these specific effects may favour or limit ROM. Yet, we observed an immediate increase in ROM in both limbs after the application of the PTR. To be noted, all the applied interventions were statistically equivalent, however the PNF stretching showed the greatest increases in absolute terms from T0 to T1 (SS > 5.75° [6.4%]; PNF > 8.6° [9.5%]; PTR > 4.85° [5.4%]), while being the only one showing a decremental trend from T1 to T2 (SS > 0.3° [0.3%]; PNF < 0.3° [− 0.35%]; PTR > 0.55° [0.6%]). Results consistent to those reported by Behm et al. in a systematic review evaluating the acute effects of different stretching typologies on ROM [5]. Future studies should also consider the time course of these techniques incorporating 30 m or 60 m post-intervention assessments.

Pertaining to jumping performance we expected a reduction in the observed values following the two stretching interventions [61–64]. These reductions, however, were not present. A careful analysis of the available scientific literature has emphasized that vertical jumps are more frequently employed as a performance post-stretching measure. Only the study of Merino-Marban et al. [65] published in 2021, similarly to our investigation, evaluated the acute post-stretching effects of long jump performance. Regardless of the differences in the analysed populations (being that primary school children were employed), the authors did not evince significant differences after the SS intervention. Differences in the biomechanical contribution of each joint are present between horizontal and vertical jumps [66, 67].Therefore, a difference in jumping technique could have determined differences in the observed outcomes across studies.

Another point to be considered is that our intervention intended to target the hamstring muscles. Such factor could be an additional element to consider affecting jumping performance [68], since the hamstrings, despite being crucial during the propulsion phase, are not the main muscular contributors of jumps [69]. However, according to Robertson et al. and Kotsifaki et al. [66, 67] the hip and knee (which are both affected by the hamstrings) contribute for ⁓ 50% to 60% of the total work during horizontal jumps.

Results from previous investigations on the effects of MFR on jumping performance provide disagreeing evidence, highlighting that MFR can either enhance it or be similar to dynamic warm ups, but could also result in a performance reduction if applied for more than 1 min [70–72]. In addition, as described in the previous paragraph, also in MFR studies vertical jumps are more frequently employed to assess lower limb performance. Only the studies of Itotani et al. [37] and Queiroga et al. [73] evaluated the effects of MFR (using two different approaches) on long jump performance. In the study of Itotani et al., the authors evaluated the immediate and post-5 days effects of continuous MFR. Despite increased jumping measures being present after 5 days, no immediate post-MFR effect was observed. Whereas Quieroga et al., applied self MFR immediately before a horizontal jump and the evaluation of different measures of ROM. Although the general increases in measures of ROM, no difference in the horizontal jump performance was observed. The results of both studies are similar to those observed in the present investigation following the PTR intervention. However, the paucity of published literature regarding both effects of stretching and MFR on long jump performance prevents us to infer definitive conclusions.

Long bouts of static stretching (greater than 45 s duration) have been observed in several investigations to acutely impair strength [25, 74], a reduction which could also affect one’s ability to balance [16, 75]. However, no univocal conclusions have to date been drawn. The increased stretch-induced joint position [74] and the reduced tendon-unit stiffness [76] could be factors increasing balance ability through proprioceptive feedback after stretching. Conversely, the reduction in neural drive post-stretching [19] could determine reductions in balance. In addition, other factors such as the previous training experience of participants or differences in assessment procedures can possibly explain the differences observed across studies. A recent study by Coratella et al. [26] has evaluated the effects of passive stretching on dynamic balance performance and muscle efficiency. While no significant differences were observed in measures of either static and dynamic balance, the authors report decreased maximal voluntary muscle contraction and decreased muscle activation, underlining a reduction in muscle efficiency. However, we did not observe a reduction in measures of balance after the SS intervention. Since we used relatively short stretch durations (30 s) and our population comprised young healthy individuals, it is plausible that if a reduction in muscle efficiency, as suggested by Coratella et al., was present, this did not transfer to a reduction in balance, being that balance is a multifactorial ability [77]. In addition, different outcomes also emerge when considering the effects of PNF on balance. The majority of the investigations carried out on healthy subjects, observe increases in both static and dynamic balance parameters [78–80]. These are particularly evident pertaining to dynamic balance in the medio-lateral direction [81, 82]. Results are in contrast to those observed in this investigation since neither for the PNF stretching we observed any effect on balance. However, these investigations applied the PNF stretching to multiple muscles (i.e. adductors and abductors, or hip flexors and extensors or all muscles of the lower limb). Therefore, the positive effects observed may not necessarily reflect the stretching phase of the PNF, but rather the contraction, which could have determined increased strength and therefore increased ability to balance [83]. Indeed, the only study applying PNF to a single group of muscles, more specifically the hamstrings (as in the present investigation), observed no increase in balance in neither the anterior–posterior nor medio-lateral directions [84]. No effects on balance were also observed after the application of the PTR technique. Results that seem to be in line with other studies investigating balance after the application of MFT techniques [39, 85]. However, it is important to stress that not many studies have been carried out on such a specific topic and being that a wide range of techniques may fall under the concept of MFR and a large heterogeneity of balance assessment procedures are adopted, it is very difficult to compare our results with those of other studies. In particular, most of the studies evaluating the effects of MFR tend to use foam rollers or carry out self-MFR interventions. Differently, the PTR technique implemented in the present investigation was manually applied by an investigator. In addition, studies may evaluate static rather than dynamic balance or adopt procedures different from the Y-balance included in the present investigation.

The present manuscript is not without limitations. Despite statistical power being reached, this is the first intervention proposing the PTR as a method for ROM improvement. Therefore, a broader body of evidence is needed to confirm our results. In addition, we adopted a research model which only accounts for intra-individual differences.

A further aspect which warrants attention is that the number of PTR stimuli (1 or 2) was determined according to the operator’s perception of the hamstrings tone. Also, the PTR differently from stretching cannot be self-administered. Therefore, coaches or therapists need to be familiar with the correct execution of the technique. However, the application of the techniques by a single operator within this study was intended to limited inter-operator differences. Despite the above discussed limitations, our results confirm the effects of PTR on ROM and are in line with other studies regarding jumping performance and balance.

The results of the present study, which arise from a population of healthy active participants, highlight a potentiality for the use of the PTR, with outcomes similar to SS and PNF. It is interesting to note that both stretching protocols (8 sets of 30 s with 30 s rest) needed at least 8 min to be carried out, while the PTR only required one to two stimuli to elicit a measurable response. Future perspectives will be to evaluate the feasibility and effectiveness of the PTR in clinical settings. If our results will be confirmed, the advantage would be to have a form of therapy which could be applied quickly and limit pain which might arise during longer forms of treatments.

## Conclusion

The present study indicates that the three proposed interventions are equivalent pertaining to the acute effects on range of movement, jumping performance and dynamic balance. Increased range of movement and no detrimental effects on performance and balance were observed. The positional transversal release technique is, however, less time-consuming compared to static stretching or proprioceptive neuromuscular facilitation, which could represent an advantage in acute settings. Despite these preliminary results, future interventions need to confirm these findings.

## Data Availability

The datasets used and/or analysed during the current study are available from the corresponding author on reasonable request.

## Abbreviations

ANOVA: Analysis of variance
CG: Control intervention
IPAQ: International Physical Activity Questionnaire
MFR: Myofascial release
MTJ: Myotendinous junction
PIC: Persistent inward current
PNF: Proprioceptive neuromuscular facilitation
PSLR: Passive straight leg raise
PTR: Positional transversal release
ROM: Range of movement
SBJ: Standing broad jump
SS: Static stretching
YBT: Y-balance test
